# Supplementary material for: The Small RNA Universe of Capitella teleta
Source: Front Mol Biosci. 2022 Feb 25;9:802814. doi: 10.3389/fmolb.2022.802814 (PMC8915122; doi:10.3389/fmolb.2022.802814)
Supplement: Supplementary file 1 [file DataSheet1.ZIP › Supplement/known/CAPTEscaffold_114_8207.pdf]

Provisional ID	:	CAPTEscaffold_114_8207
Score total	:	2095954.6
Score for star read(s)	:	3.9
Score for read counts	:	2095944.7
Score for mfe	:	1.3
Score for randfold	:	1.6
Score for cons. seed	:	3
Total read count	:	4111117
Mature read count	:	4111111
Loop read count	:	0
Star read count	:	6

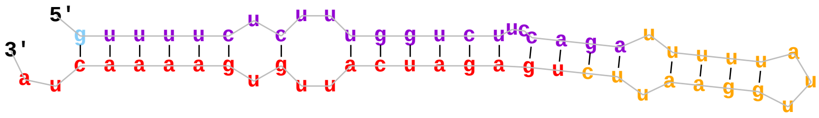

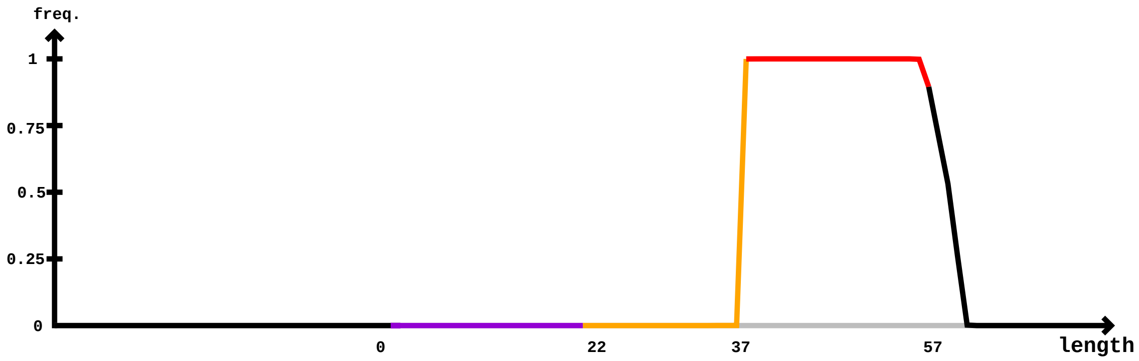


```
                                                   Star                                  Mature
5'- ugcgaauguuucagagcaagacuugcgagacugguuuucucuuuggucuuccagauuuuuauuggaauucugagaucauugugaaaacuaaucucgcugacauuacgc  -3'   obs
    ugcgaauguuucagagcaagacuugcgagacugguuuucucuuuggucuuccagauuuuuauuggaauucugagaucauugugaaaacuaaucucgcugacauuacgc        exp
    ugcgaauguuucagagcaagacuugcgagacugguuuucucuuuggucuuccagauuuuuauuggaauucugagaucauugugaaaacuaaucucgcugacauuacgc        known
    .(((((((((..............((((((.((((((((.(..((((((..((((.((((...)))).))))))))))..).)))))))).)))))).)))))).)))   reads   mm   sample
    ..............................cugguuuucucuuuggucuu..........................................................   1       0    seq
    ..............................cugguuuucucuuuggucuuc.........................................................   2       0    seq
    ..............................cugguuuucucuuuAgucuucc........................................................   1       1    seq
    ..............................cugguuuucucuuuggucuucc........................................................   31      0    seq
    ..............................cugguuuucucuuuggucuucca.......................................................   29      0    seq
    ..............................cugguuuucucuuuggucuuccU.......................................................   3       1    seq
    ..............................cugguuuucucuuuggucuuccag......................................................   1       0    seq
    ..............................cugguuuucucuuuggucuuccaga.....................................................   49      0    seq
    ..............................cuAguuuucucuuuggucuuccaga.....................................................   1       1    seq
    .................................guuuucucuuuggucuuccaga.....................................................   1       0    seq
    ..................................uuuucucuuuggucuuccaga.....................................................   3       0    seq
    ..................................uuuucucuuuggucuuccagauu...................................................   2       0    seq
    ...................................................................uucugagaucauugugaaa......................   18      0    seq
    ...................................................................uucugagaucauugugaaaa.....................   7       0    seq
    ...................................................................uucugagaucauugugaaaac....................   6       0    seq
    ...................................................................Nucugagaucauugugaaaacu...................   1       1    seq
    ...................................................................Aucugagaucauugugaaaacu...................   7       1    seq
    ...................................................................uucugagaucauugugaaaUcu...................   1       1    seq
    ...................................................................uucugagaucaAugugaaaacu...................   1       1    seq
    ...................................................................uucugagaucauuguAaaaacu...................   2       1    seq
    ...................................................................uucugagaucauugugGaaacu...................   1       1    seq
    ...................................................................uucugagaucauugugaaaacu...................   1232    0    seq
    ...................................................................uucAgagaucauugugaaaacu...................   1       1    seq
    ...................................................................uucugagaucauugugaaaacG...................   1       1    seq
    ...................................................................uucugagaucauugCgaaaacu...................   1       1    seq
    ...................................................................uucugagUucauugugaaaacu...................   1       1    seq
    ...................................................................Gucugagaucauugugaaaacu...................   1       1    seq
    ...................................................................uucugagaucauugugaaaaAu...................   1       1    seq
    ...................................................................uucugagaucauugugaaaacC...................   4       1    seq
    ...................................................................uuUugagaucauugugaaaacu...................   1       1    seq
    ...................................................................uucugagaucauugugaaaacua..................   3       0    seq
    ...................................................................uucugagaucauugugaaaacuU..................   1       1    seq
```

Star Mature

ugcgaauguuucagagcaagacuugcgagacugguuuucucuuuggucuuccagauuuuuauuggaauucugagaucauugugaaaacuaaucucgcugacauuacgc			
....ucugagaucauugugaaaacu...................	6	0	seq
....ucugagaucauugugaaaacua..................	3	0	seq
....cAgagaucauugugaaaacu...................	1	1	seq
....Uugagaucauugugaaaacu...................	1	1	seq
....cugagaucauugugaaaacu...................	27	0	seq
....cugagaucauugugaaaacua..................	7	0	seq
....cugagaucauugugaaaacuaa.................	2	0	seq
....Augagaucauugugaaaacuaa.................	1	1	seq
....cugagaucauugugaaaacuaCu................	2	1	seq
....cugagaucauugugaaaacuaau................	2	0	seq
....Gugagaucauugugaaaacuaau................	1	1	seq
....Augagaucauugugaaaacuaau................	20	1	seq
....Gugagaucauugugaaaacuaauc...............	1	1	seq
....cugagaucauugugaaaacuaauc...............	15	0	seq
....ugagaucauugugUaaac....................	1	1	seq
....uCagaucauugugaaaac....................	1	1	seq
....ugaAaucauugugaaaac....................	2	1	seq
....ugagauGauugugaaaac....................	2	1	seq
....Cgagaucauugugaaaac....................	3	1	seq
....uAagaucauugugaaaac....................	4	1	seq
....Agagaucauugugaaaac....................	29	1	seq
....ugagaAcauugugaaaac....................	1	1	seq
....ugagaucauugCgaaaac....................	2	1	seq
....ugagaucauGgugaaaac....................	100	1	seq
....Ggagaucauugugaaaac....................	5	1	seq
....ugagaucauCgugaaaac....................	3	1	seq
....ugagaucauugugGaaac....................	2	1	seq
....ugagaucauAgugaaaac....................	5	1	seq
....ugagaucauuguAaaaac....................	6	1	seq
....ugUgaucauugugaaaac....................	1	1	seq
....ugagaucaCugugaaaac....................	1	1	seq
....ugagauAauugugaaaac....................	8	1	seq
....ugagaucaGugugaaaac....................	2	1	seq
....ugagaucauugugaaaaU....................	132	1	seq
....ugagaucauugugaaaGc....................	1	1	seq
....ugagaucauugugaaaaA....................	623	1	seq
....ugagaucauugugaaaaG....................	34	1	seq
....ugagaucauugugaaaCc....................	1	1	seq
....ugagUucauugugaaaac....................	1	1	seq
....ugagaucauuAugaaaac....................	2	1	seq
....ugagaucauuguUaaaac....................	1	1	seq
....Ngagaucauugugaaaac....................	1	1	seq
....ugagaucauugugaaGac....................	1	1	seq
....ugagaucauugAgaaaac....................	4	1	seq
....ugagaucauugugaaaac....................	5154	0	seq
....ugagaucauugugaGaacu...................	59	1	seq
....ugagaucGuugugaaaacu...................	48	1	seq
....uCagaucauugugaaaacu...................	74	1	seq
....ugagaucauuUugaaaacu...................	29	1	seq
....ugagaucauugugaaaaAu...................	43	1	seq
....uAagaucauugugaaaacu...................	300	1	seq
....ugagaucauugGgaaaacu...................	24	1	seq
....ugagaAcauugugaaaacu...................	99	1	seq
....ugagGucauugugaaaacu...................	61	1	seq
....ugagaucauugugaaaacu...................	415487	0	seq
....uNagaucauugugaaaacu...................	9	1	seq
....ugagaucauuAugaaaacu...................	78	1	seq
....ugagaucauCgugaaaacu...................	69	1	seq
....ugagaucauugugUaaacu...................	57	1	seq
....ugaCaucauugugaaaacu...................	15	1	seq
....ugNgaucauugugaaaacu...................	2	1	seq
....ugaAaucauugugaaaacu...................	72	1	seq
....ugagauGauugugaaaacu...................	35	1	seq
....Cgagaucauugugaaaacu...................	104	1	seq
....ugagaucauugugNaaacu...................	1	1	seq
....ugagaucauuguUaaaacu...................	33	1	seq
....ugagaucauugugaaaaGu...................	8	1	seq
....ugagaucauugugaaGacu...................	68	1	seq
....Ngagaucauugugaaaacu...................	142	1	seq
....ugagaucaCugugaaaacu...................	35	1	seq

Star Mature

ugcgaauguuucagagcaagacuugcgagacugguuuucucuuuggucuuccagauuuuuauuggaauucugagaucauugugaaaacuaaucucgcugacauuacgc			
....ugagaucauugugaaaacA...................	128	1	seq
....ugagaucauugugaaaacG...................	919	1	seq
....ugagauAauugugaaaacu...................	618	1	seq
....ugagUucauugugaaaacu...................	52	1	seq
....ugagCucauugugaaaacu...................	6	1	seq
....ugaUaucauugugaaaacu...................	21	1	seq
....ugagaucauugCgaaaacu...................	68	1	seq
....ugagaucauugugGaaacu...................	73	1	seq
....ugagaucauugugaaaCcu...................	53	1	seq
....Ggagaucauugugaaaacu...................	210	1	seq
....ugagaucauugugaUaacu...................	12	1	seq
....ugagaucaGugugaaaacu...................	27	1	seq
....ugagaucauAgugaaaacu...................	338	1	seq
....ugagaucauugugaaCacu...................	3	1	seq
....ugCgaucauugugaaaacu...................	3	1	seq
....ugagaucauugugaaaUcu...................	46	1	seq
....ugagaucaNugugaaaacu...................	1	1	seq
....ugagaucauuguCaaaacu...................	33	1	seq
....ugagaucUuugugaaaacu...................	36	1	seq
....ugagauUauugugaaaacu...................	232	1	seq
....Agagaucauugugaaaacu...................	1991	1	seq
....uUagaucauugugaaaacu...................	64	1	seq
....ugUgaucauugugaaaacu...................	37	1	seq
....ugagaucauGgugaaaacu...................	195	1	seq
....ugagaucauugugaaaNcu...................	2	1	seq
....ugagaucauugugCaaacu...................	4	1	seq
....ugagaucauuCugaaaacu...................	23	1	seq
....ugagaucCuugugaaaacu...................	3	1	seq
....ugagaucauugAgaaaacu...................	237	1	seq
....ugagaucauugugaaaacC...................	46	1	seq
....ugGgaucauugugaaaacu...................	61	1	seq
....ugagaucauugugaaaGcu...................	54	1	seq
....ugagaGcauugugaaaacu...................	8	1	seq
....ugagaucauugugaaaaUu...................	31	1	seq
....ugagaucauuguAaaaacu...................	659	1	seq
....ugagaNcauugugaaaacu...................	6	1	seq
....ugagaCcauugugaaaacu...................	48	1	seq
....ugagaucauuNugaaaacu...................	1	1	seq
....ugagaucauugugaaUacu...................	16	1	seq
....ugagaucauugugaaaacuC..................	22	1	seq
....ugagaucauugugaaaGcua..................	155	1	seq
....ugagaucauugugaCaacua..................	8	1	seq
....ugagaucauugugaaaacuU..................	2888	1	seq
....ugagauGauugugaaaacua..................	97	1	seq
....ugagaucUuugugaaaacua..................	111	1	seq
....ugagaucauAgugaaaacua..................	891	1	seq
....ugagaucauugugaaGacua..................	168	1	seq
....ugagaucauugugaaaaGua..................	30	1	seq
....ugagaucauugGgaaaacua..................	45	1	seq
....ugNgaucauugugaaaacua..................	1	1	seq
....ugagaucauGgugaaaacua..................	24	1	seq
....ugagaucauugugaaaNcua..................	4	1	seq
....ugagaucauugugGaaacua..................	201	1	seq
....ugagaucauuCugaaaacua..................	55	1	seq
....Agagaucauugugaaaacua..................	5770	1	seq
....ugagaucauugugaaaCcua..................	138	1	seq
....ugagaucauugugaUaacua..................	42	1	seq
....ugagaucauuguUaaaacua..................	95	1	seq
....ugagaucauugugCaaacua..................	15	1	seq
....ugGgaucauugugaaaacua..................	167	1	seq
....ugagaGcauugugaaaacua..................	25	1	seq
....ugUgaucauugugaaaacua..................	93	1	seq
....ugagaucauuUugaaaacua..................	73	1	seq
....ugagaucauugugaaaaUua..................	65	1	seq
....uCagaucauugugaaaacua..................	209	1	seq
....ugagaucauugugaaaacuN..................	2	1	seq
....ugagaucauugugaGaacua..................	199	1	seq
....ugagaucaGugugaaaacua..................	66	1	seq
....ugCgaucauugugaaaacua..................	12	1	seq
....ugaAaucauugugaaaacua..................	159	1	seq

```
                                  Star                                Mature
ugcgaauguuucagagcaagacuugcgagacugguuuucucuuuggucuuccagauuuuuauuggaauucugagaucauugugaaaacuaaucucgcugacauuacgc
......................................................................ugagauUauugugaaaacua..................   648      1    seq
......................................................................ugagaucauugAgaaaacua..................   715      1    seq
......................................................................ugagaucCuugugaaaacua..................   6        1    seq
......................................................................ugagaucauugugNaaacua..................   2        1    seq
......................................................................ugagaucaCugugaaaacua..................   135      1    seq
......................................................................ugagaucauugugaaaUcua..................   218      1    seq
......................................................................ugagaucauugugaaaacCa..................   163      1    seq
......................................................................ugagaucaAugugaaaacua..................   213      1    seq
......................................................................uUagaucauugugaaaacua..................   181      1    seq
......................................................................ugagGucauugugaaaacua..................   166      1    seq
......................................................................ugagaNcauugugaaaacua..................   24       1    seq
......................................................................Ggagaucauugugaaaacua..................   678      1    seq
......................................................................ugagaucauNgugaaaacua..................   6        1    seq
......................................................................Cgagaucauugugaaaacua..................   264      1    seq
......................................................................ugagaucauuAugaaaacua..................   222      1    seq
......................................................................ugaUaucauugugaaaacua..................   64       1    seq
......................................................................ugagauAauugugaaaacua..................   1538     1    seq
......................................................................ugagaAcauugugaaaacua..................   315      1    seq
......................................................................ugagaucauugugaaaacuG..................   901      1    seq
......................................................................ugagaucauuguAaaaacua..................   1875     1    seq
......................................................................ugagaucGuugugaaaacua..................   149      1    seq
......................................................................ugagaucauugugaaUacua..................   80       1    seq
......................................................................ugagaucauugugUaaacua..................   112      1    seq
......................................................................ugagaucauugugaaaacAa..................   151      1    seq
......................................................................ugagaucauugugaaCacua..................   5        1    seq
......................................................................ugagCucauugugaaaacua..................   14       1    seq
......................................................................ugagUucauugugaaaacua..................   138      1    seq
......................................................................ugagaucauugugaaaacua..................   1197321  0    seq
......................................................................Ngagaucauugugaaaacua..................   370      1    seq
......................................................................ugaCaucauugugaaaacua..................   45       1    seq
......................................................................ugagaucauugCgaaaacua..................   184      1    seq
......................................................................ugagaucauCgugaaaacua..................   187      1    seq
......................................................................uNagaucauugugaaaacua..................   31       1    seq
......................................................................uAagaucauugugaaaacua..................   943      1    seq
......................................................................ugagaCcauugugaaaacua..................   104      1    seq
......................................................................ugagaucauuguCaaaacua..................   87       1    seq
......................................................................ugagaucauugugaaaacGa..................   56       1    seq
......................................................................ugagaucauugugaaaaAua..................   77       1    seq
......................................................................ugagaucauugugaaCacuaa.................   2        1    seq
......................................................................Agagaucauugugaaaacuaa.................   1203     1    seq
......................................................................ugagaucauugugaaaaUuaa.................   20       1    seq
......................................................................ugagaucauuNugaaaacuaa.................   1        1    seq
......................................................................ugagaucUuugugaaaacuaa.................   29       1    seq
......................................................................ugagaucauugugaaaCcuaa.................   28       1    seq
......................................................................ugagaucauugugaaaacuNa.................   1        1    seq
......................................................................ugUgaucauugugaaaacuaa.................   29       1    seq
......................................................................uCagaucauugugaaaacuaa.................   36       1    seq
......................................................................Ngagaucauugugaaaacuaa.................   86       1    seq
......................................................................ugagaucauugugGaaacuaa.................   45       1    seq
......................................................................ugagaucauuguCaaaacuaa.................   16       1    seq
......................................................................ugagaucauuUugaaaacuaa.................   23       1    seq
......................................................................ugaCaucauugugaaaacuaa.................   10       1    seq
......................................................................ugagaucauugAgaaaacuaa.................   169      1    seq
......................................................................ugagaGcauugugaaaacuaa.................   4        1    seq
......................................................................ugagaucauugNgaaaacuaa.................   1        1    seq
......................................................................ugaUaucauugugaaaacuaa.................   18       1    seq
......................................................................ugagaucauugugaaaacuGa.................   63       1    seq
......................................................................ugaAaucauugugaaaacuaa.................   33       1    seq
......................................................................uUagaucauugugaaaacuaa.................   36       1    seq
......................................................................ugagaucaAugugaaaacuaa.................   45       1    seq
......................................................................ugagaucGuugugaaaacuaa.................   33       1    seq
......................................................................uNagaucauugugaaaacuaa.................   6        1    seq
......................................................................ugagaucauCgugaaaacuaa.................   33       1    seq
......................................................................ugagaucauugugaaaacuaG.................   81       1    seq
......................................................................ugagaucCuugugaaaacuaa.................   1        1    seq
......................................................................ugagaucauugugCaaacuaa.................   1        1    seq
......................................................................ugagaAcauugugaaaacuaa.................   79       1    seq
......................................................................ugagaucauuguAaaaacuaa.................   475      1    seq
......................................................................uAagaucauugugaaaacuaa.................   183      1    seq
......................................................................ugagaucauugugaaaUcuaa.................   46       1    seq
```

```
                                  Star                                 Mature
ugcgaauguuucagagcaagacuugcgagacugguuuucucuuuggucuuccagauuuuuauuggaauucugagaucauugugaaaacuaaaucucgcugacauuacgc
......................................................................ugagaucauugGgaaaacuaa.................   20       1    seq
......................................................................ugagaucauugugaaGacuaa.................   47       1    seq
......................................................................ugagaucauugugUaaacuaa.................   21       1    seq
......................................................................ugagauUauugugaaaacuaa.................   151      1    seq
......................................................................Cgagaucauugugaaaacuaa.................   60       1    seq
......................................................................ugagaucauAgugaaaacuaa.................   174      1    seq
......................................................................ugCgaucauugugaaaacuaa.................   10       1    seq
......................................................................ugagCucauugugaaaacuaa.................   3        1    seq
......................................................................ugagaucaGugugaaaacuaa.................   19       1    seq
......................................................................ugagaucauuCugaaaacuaa.................   16       1    seq
......................................................................ugagaucauuAugaaaacuaa.................   43       1    seq
......................................................................ugagaucauugugaaaacuaN.................   2        1    seq
......................................................................ugagaucauugugaaaacCaa.................   27       1    seq
......................................................................ugagUucauugugaaaacuaa.................   36       1    seq
......................................................................Ggagaucauugugaaaacuaa.................   178      1    seq
......................................................................ugNgaucauugugaaaacuaa.................   2        1    seq
......................................................................ugagaucaCugugaaaacuaa.................   25       1    seq
......................................................................ugagaCcauugugaaaacuaa.................   19       1    seq
......................................................................ugagaucauugugaaaNcuaa.................   1        1    seq
......................................................................ugagaucauugugaaaacuUa.................   24       1    seq
......................................................................ugagauAauugugaaaacuaa.................   366      1    seq
......................................................................ugagaNcauugugaaaacuaa.................   5        1    seq
......................................................................ugagauGauugugaaaacuaa.................   16       1    seq
......................................................................ugagaucauuguUaaaacuaa.................   18       1    seq
......................................................................ugagaucauNgugaaaacuaa.................   1        1    seq
......................................................................ugagGucauugugaaaacuaa.................   41       1    seq
......................................................................ugagaucauugugaaUacuaa.................   19       1    seq
......................................................................ugagaucauGgugaaaacuaa.................   10       1    seq
......................................................................ugagaucauugugaaaGcuaa.................   29       1    seq
......................................................................ugagaucauugugaaaaGuaa.................   8        1    seq
......................................................................ugagaucauugugaaaacuaC.................   5        1    seq
......................................................................ugagaucauugugaUaacuaa.................   13       1    seq
......................................................................ugagaucauugugaaaaAuaa.................   17       1    seq
......................................................................ugagaucauugugaaNacuaa.................   1        1    seq
......................................................................ugagaucauugugaaaacAaa.................   37       1    seq
......................................................................ugagaucauugugaaaacuaU.................   991      1    seq
......................................................................ugagaucauugugaGaacuaa.................   44       1    seq
......................................................................ugagaucauugugaaaacuaa.................   269268   0    seq
......................................................................ugagaucauugCgaaaacuaa.................   47       1    seq
......................................................................ugGgaucauugugaaaacuaa.................   35       1    seq
......................................................................ugagaucauugugaaaacGaa.................   9        1    seq
......................................................................ugagaucauugAgaaaacuaau................   672      1    seq
......................................................................uNagaucauugugaaaacuaau................   34       1    seq
......................................................................ugagaucaCugugaaaacuaau................   120      1    seq
......................................................................ugagaucauugugaaaacuaaA................   5033     1    seq
......................................................................ugagaucauugugaaaaAuaau................   104      1    seq
......................................................................ugagaucauugugaaaacuaaG................   185      1    seq
......................................................................ugagaucCuugugaaaacuaau................   10       1    seq
......................................................................ugagauAauugugaaaacuaau................   1291     1    seq
......................................................................ugaUaucauugugaaaacuaau................   76       1    seq
......................................................................ugagauGauugugaaaacuaau................   109      1    seq
......................................................................ugagaucauugugaaUacuaau................   94       1    seq
......................................................................ugagaucauugugaaaaUuaau................   84       1    seq
......................................................................ugagaucauCgugaaaacuaau................   161      1    seq
......................................................................ugGgaucauugugaaaacuaau................   169      1    seq
......................................................................Ggagaucauugugaaaacuaau................   636      1    seq
......................................................................ugagauUauugugaaaacuaau................   756      1    seq
......................................................................ugagaCcauugugaaaacuaau................   79       1    seq
......................................................................ugagaucauugugaaaacuUau................   34       1    seq
......................................................................ugagaucauugugGaaacuaau................   192      1    seq
......................................................................ugagaucaAugugaaaacuaau................   240      1    seq
......................................................................ugagaucauugugaaaacuaau................   1089751  0    seq
......................................................................uAagaucauugugaaaacuaau................   1079     1    seq
......................................................................ugagaucauugugaaCacuaau................   11       1    seq
......................................................................ugagaucUuugugaaaacuaau................   127      1    seq
......................................................................ugagaucauugugaaaacuaaC................   82       1    seq
......................................................................ugagaucauGgugaaaacuaau................   18       1    seq
......................................................................ugagaucGuugugaaaacuaau................   142      1    seq
......................................................................ugagaucauugugaaaacuaUu................   102      1    seq
......................................................................Agagaucauugugaaaacuaau................   5028     1    seq
```

Star **Mature**

```
ugcgaauguuucagagcaagacuugcgagacugguuuucucuuuggucuuccagauuuuuauuggaauucugagaucauugugaaaacuaaucucgcugacauuacgc
......................................................................ugagaucauugugaaaCcuaau................ 120      1    seq
......................................................................Cgagaucauugugaaaacuaau................ 266      1    seq
......................................................................ugaAaucauugugaaaacuaau................ 139      1    seq
......................................................................ugagaucauugGgaaaacuaau................ 72       1    seq
......................................................................ugagaucauugugaCaacuaau................ 3        1    seq
......................................................................ugagaucaGugugaaaacuaau................ 66       1    seq
......................................................................ugagaucauugugaaaacuaaN................ 1        1    seq
......................................................................ugagaGcauugugaaaacuaau................ 15       1    seq
......................................................................ugagaucauuguUaaaacuaau................ 95       1    seq
......................................................................ugagaucauugugaaaacGaau................ 52       1    seq
......................................................................ugagaucauugugCaaacuaau................ 8        1    seq
......................................................................ugagaucauuAugaaaacuaau................ 196      1    seq
......................................................................ugagaucauugugaaaacCaau................ 128      1    seq
......................................................................ugagaucauugugaaaacuGau................ 236      1    seq
......................................................................ugagCucauugugaaaacuaau................ 14       1    seq
......................................................................ugagaucauugCgaaaacuaau................ 205      1    seq
......................................................................ugagaucauuNugaaaacuaau................ 1        1    seq
......................................................................ugagaucauuguCaaaacuaau................ 85       1    seq
......................................................................ugagGucauugugaaaacuaau................ 154      1    seq
......................................................................Ngagaucauugugaaaacuaau................ 371      1    seq
......................................................................ugagaucauugugaaaacuaNu................ 7        1    seq
......................................................................ugagaucauugugaaaaGuaau................ 35       1    seq
......................................................................ugagaucauugugUaaacuaau................ 143      1    seq
......................................................................ugNgaucauugugaaaacuaau................ 2        1    seq
......................................................................ugagaucauuCugaaaacuaau................ 50       1    seq
......................................................................ugCgaucauugugaaaacuaau................ 17       1    seq
......................................................................ugagaucauugugaaaacuCau................ 3        1    seq
......................................................................ugagaucauugugaaaUcuaau................ 164      1    seq
......................................................................ugagaucauugugaaaacuaGu................ 133      1    seq
......................................................................ugagaNcauugugaaaacuaau................ 12       1    seq
......................................................................ugagUucauugugaaaacuaau................ 142      1    seq
......................................................................ugagaucauuUugaaaacuaau................ 92       1    seq
......................................................................ugagaucauuguAaaaacuaau................ 1889     1    seq
......................................................................ugagaAcauugugaaaacuaau................ 310      1    seq
......................................................................uCagaucauugugaaaacuaau................ 170      1    seq
......................................................................ugagaucauugugaaaacAaau................ 163      1    seq
......................................................................ugaCaucauugugaaaacuaau................ 83       1    seq
......................................................................ugagaucauNgugaaaacuaau................ 3        1    seq
......................................................................ugagaucauugugaaGacuaau................ 193      1    seq
......................................................................ugUgaucauugugaaaacuaau................ 82       1    seq
......................................................................ugagaucauugugaaaGcuaau................ 153      1    seq
......................................................................ugagaucauugugNaaacuaau................ 1        1    seq
......................................................................ugagaucauugugaaaacNaau................ 1        1    seq
......................................................................ugagaucauugugaUaacuaau................ 81       1    seq
......................................................................ugagaucauugugaaaacuaCu................ 21       1    seq
......................................................................ugagaucauugugaGaacuaau................ 175      1    seq
......................................................................ugagaucauAgugaaaacuaau................ 715      1    seq
......................................................................uUagaucauugugaaaacuaau................ 183      1    seq
......................................................................ugagaucauugugaaaacuUauc............... 18       1    seq
......................................................................ugagaAcauugugaaaacuaauc............... 289      1    seq
......................................................................ugagaucauCgugaaaacuaauc............... 208      1    seq
......................................................................Agagaucauugugaaaacuaauc............... 4760     1    seq
......................................................................ugagaucauuAugaaaacuaauc............... 194      1    seq
......................................................................uNagaucauugugaaaacuaauc............... 32       1    seq
......................................................................ugagaucauugugUaaacuaauc............... 126      1    seq
......................................................................ugUgaucauugugaaaacuaauc............... 95       1    seq
......................................................................ugagaucauugugaaCacuaauc............... 9        1    seq
......................................................................ugagaucauugugaaaacuaauN............... 7        1    seq
......................................................................ugagaucauugugaaNacuaauc............... 1        1    seq
......................................................................ugagaucauugCgaaaacuaauc............... 194      1    seq
......................................................................ugagaucauugugaaaacuaauc............... 1040595  0    seq
......................................................................ugagaucauugAgaaaacuaauc............... 615      1    seq
......................................................................ugagaucauugGgaaaacuaauc............... 52       1    seq
......................................................................ugagaucaAugugaaaacuaauc............... 191      1    seq
......................................................................ugagaucauuguUaaaacuaauc............... 90       1    seq
......................................................................ugGgaucauugugaaaacuaauc............... 126      1    seq
......................................................................uUagaucauugugaaaacuaauc............... 158      1    seq
......................................................................ugagaucauuCugaaaacuaauc............... 46       1    seq
......................................................................uAagaucauugugaaaacuaauc............... 1260     1    seq
......................................................................ugagaucauugugCaaacuaauc............... 4        1    seq
```

Star Mature

```
ugcgaauguuucagagcaagacuugcgagacugguuuucucuuuggucuuccagauuuuuauuggaauucugagaucauugugaaaacuaaucucgcugacauuacgc
......................................................................ugagauUauugugaaaacuaauc............... 935 1 seq
......................................................................ugCgaucauugugaaaacuaauc............... 18 1 seq
......................................................................ugagaucaGugugaaaacuaauc............... 56 1 seq
......................................................................ugagaNcauugugaaaacuaauc............... 10 1 seq
......................................................................ugagaucauugugaaaacCaauc............... 116 1 seq
......................................................................uCagaucauugugaaaacuaauc............... 162 1 seq
......................................................................ugagaucGuugugaaaacuaauc............... 127 1 seq
......................................................................ugagaucauugugaaaacuaCuc............... 6 1 seq
......................................................................ugagaucUuugugaaaacuaauc............... 99 1 seq
......................................................................ugagaucauugugaaaGcuaauc............... 138 1 seq
......................................................................ugagUucauugugaaaacuaauc............... 141 1 seq
......................................................................ugaNaucauugugaaaacuaauc............... 1 1 seq
......................................................................ugagaucauugugaaaacuaauG............... 167 1 seq
......................................................................ugagaucaCugugaaaacuaauc............... 114 1 seq
......................................................................ugNgaucauugugaaaacuaauc............... 5 1 seq
......................................................................ugagaucauugugaaaacuaaCc............... 82 1 seq
......................................................................ugagaucauugugaaaacuGauc............... 295 1 seq
......................................................................ugagaucauugugaaaaGuaauc............... 26 1 seq
......................................................................ugagaucauGgugaaaacuaauc............... 25 1 seq
......................................................................ugagaucauugugaCaacuaauc............... 5 1 seq
......................................................................ugagauGauugugaaaacuaauc............... 121 1 seq
......................................................................ugagaucauuguCaaaacuaauc............... 109 1 seq
......................................................................ugagaucauugugaaaaAuaauc............... 78 1 seq
......................................................................ugaAaucauugugaaaacuaauc............... 164 1 seq
......................................................................ugagGucauugugaaaacuaauc............... 129 1 seq
......................................................................ugagaucauuUugaaaacuaauc............... 63 1 seq
......................................................................ugagaucauugugaaaacuaUuc............... 70 1 seq
......................................................................ugagaucauugugaGaacuaauc............... 185 1 seq
......................................................................ugagaucauuNugaaaacuaauc............... 1 1 seq
......................................................................ugagaucauugugaaaacuCauc............... 8 1 seq
......................................................................ugagaucauugugaaaacuaauA............... 3076 1 seq
......................................................................ugagaucauugugaaaacuaGuc............... 140 1 seq
......................................................................ugagaucauugugaaaacuaaGc............... 27 1 seq
......................................................................ugagaCcauugugaaaacuaauc............... 92 1 seq
......................................................................ugagaucauugugaaaacuaaAc............... 283 1 seq
......................................................................ugagaucCuugugaaaacuaauc............... 7 1 seq
......................................................................ugagaucauugugaaaUcuaauc............... 178 1 seq
......................................................................ugagaucauuguAaaaacuaauc............... 2748 1 seq
......................................................................ugagaucauugugaaaacuaauU............... 976 1 seq
......................................................................Ggagaucauugugaaaacuaauc............... 617 1 seq
......................................................................ugagaucauugugaaaCcuaauc............... 118 1 seq
......................................................................Cgagaucauugugaaaacuaauc............... 253 1 seq
......................................................................ugagaGcauugugaaaacuaauc............... 21 1 seq
......................................................................ugagaucauugugaUaacuaauc............... 67 1 seq
......................................................................ugagaucauugugGaaacuaauc............... 146 1 seq
......................................................................Ngagaucauugugaaaacuaauc............... 313 1 seq
......................................................................ugagauAauugugaaaacuaauc............... 1196 1 seq
......................................................................ugaUaucauugugaaaacuaauc............... 57 1 seq
......................................................................ugagaucauugugaaaacGaauc............... 44 1 seq
......................................................................ugagaucauugugNaaacuaauc............... 1 1 seq
......................................................................ugaCaucauugugaaaacuaauc............... 41 1 seq
......................................................................ugagaucauugugaaaacuaNuc............... 5 1 seq
......................................................................ugagCucauugugaaaacuaauc............... 12 1 seq
......................................................................ugagaucauugugaaaacAaauc............... 178 1 seq
......................................................................ugagaucauAgugaaaacuaauc............... 727 1 seq
......................................................................ugagaucauugugaaUacuaauc............... 103 1 seq
......................................................................ugagaucauugugaaaaUuaauc............... 101 1 seq
......................................................................ugagaucauugugaaGacuaauc............... 168 1 seq
......................................................................ugagaucauugugaaaacuaaucC.............. 5 1 seq
......................................................................ugagUucauugugaaaacuaaucu.............. 1 1 seq
......................................................................ugaAaucauugugaaaacuaaucu.............. 2 1 seq
......................................................................ugagaucauugugaaaacuaauAu.............. 15 1 seq
......................................................................ugagaucauugugaaaacuaaAcu.............. 5 1 seq
......................................................................ugGgaucauugugaaaacuaaucu.............. 6 1 seq
......................................................................ugagGucauugugaaaacuaaucu.............. 1 1 seq
......................................................................ugagaucauugugaaaacuaaucu.............. 6418 0 seq
......................................................................ugagaucauugugaUaacuaaucu.............. 1 1 seq
......................................................................ugagaucauugugaaaGcuaaucu.............. 1 1 seq
......................................................................ugagaucauugugaaaacuaaucN.............. 6 1 seq
......................................................................ugagaucauugugaaGacuaaucu.............. 1 1 seq
```

Star **Mature**

```
ugcgaauguuucagagcaagacuugcgagacugguuuucucuuuggucuuccagauuuuuauuggaauucugagaucauugugaaaacuaaucucgcugacauuacgc
......................................................................ugagauGauugugaaaacuaaucu..............   1     1   seq
......................................................................ugagaucauuguUaaaacuaaucu..............   2     1   seq
......................................................................ugagaucauugugaaaacuaauUu..............   2     1   seq
......................................................................ugagaucauugAgaaaacuaaucu..............   3     1   seq
......................................................................ugagaucauugugaaaUcuaaucu..............   2     1   seq
......................................................................uCagaucauugugaaaacuaaucu..............   2     1   seq
......................................................................ugagauUauugugaaaacuaaucu..............   5     1   seq
......................................................................uAagaucauugugaaaacuaaucu..............   7     1   seq
......................................................................ugagaucauuguAaaaacuaaucu..............   12    1   seq
......................................................................ugagaucauugugaaaacAaaucu..............   2     1   seq
......................................................................Ggagaucauugugaaaacuaaucu..............   3     1   seq
......................................................................ugagaucauugugaGaacuaaucu..............   1     1   seq
......................................................................Ngagaucauugugaaaacuaaucu..............   5     1   seq
......................................................................ugagaucauugCgaaaacuaaucu..............   3     1   seq
......................................................................ugagaucauugugaaaCcuaaucu..............   1     1   seq
......................................................................ugagaucauugugaaaacuaaucG.............   11    1   seq
......................................................................ugagaucaCugugaaaacuaaucu..............   4     1   seq
......................................................................ugagaucauugugaaaaAuaaucu..............   1     1   seq
......................................................................ugagauAauugugaaaacuaaucu..............   9     1   seq
......................................................................ugagaucauuCugaaaacuaaucu..............   2     1   seq
......................................................................ugagaucauugugUaaacuaaucu..............   1     1   seq
......................................................................ugagaucauugugaaaacuaGucu..............   1     1   seq
......................................................................Agagaucauugugaaaacuaaucu..............   28    1   seq
......................................................................ugagaucauugugaaaacuCaucu..............   1     1   seq
......................................................................ugagaucauuguCaaaacuaaucu..............   1     1   seq
......................................................................ugagaucauAgugaaaacuaaucu..............   4     1   seq
......................................................................ugagaucauugugaaaacuaaucA.............   1078  1   seq
......................................................................ugagaucauugugaaaacuaaucuA............   1     1   seq
.......................................................................gagaAcauugugaaaacu...................   1     1   seq
.......................................................................gagaucauugugaaaaAu...................   1     1   seq
.......................................................................gagaucauugugaaaacG...................   1     1   seq
.......................................................................Uagaucauugugaaaacu...................   16    1   seq
.......................................................................gagaucauugugaaaacu...................   166   0   seq
.......................................................................gagauGauugugaaaacu...................   6     1   seq
.......................................................................gagauAauugugaaaacu...................   1     1   seq
.......................................................................gagaucauugugaaaacua..................   113   0   seq
.......................................................................gagaucauugugaaaacuU..................   1     1   seq
.......................................................................gagauGauugugaaaacua..................   2     1   seq
.......................................................................gagaucauugugaaaaAua..................   1     1   seq
.......................................................................Uagaucauugugaaaacua..................   45    1   seq
.......................................................................gagaucauugugaaaacAa..................   1     1   seq
.......................................................................Uagaucauugugaaaacuaa.................   8     1   seq
.......................................................................gagaucauugugaaaacuaa.................   19    0   seq
.......................................................................gagaucauuguAaaaacuaau................   1     1   seq
.......................................................................gagaucauugugaaaacuGau................   1     1   seq
.......................................................................gagaucauugugaaaacuaau................   115   0   seq
.......................................................................gagaucauugugaaaacAaau................   1     1   seq
.......................................................................gagaucauuUugaaaacuaau................   1     1   seq
.......................................................................gagauGauugugaaaacuaau................   3     1   seq
.......................................................................gagaucauugAgaaaacuaau................   1     1   seq
.......................................................................Uagaucauugugaaaacuaau................   39    1   seq
.......................................................................gagaucauugugaaaacuaaA................   1     1   seq
.......................................................................gagaucauugugaaaaAuaau................   1     1   seq
.......................................................................gagauGauugugaaaacuaauc...............   3     1   seq
.......................................................................Aagaucauugugaaaacuaauc...............   1     1   seq
.......................................................................gagaucauuguAaaaacuaauc...............   3     1   seq
.......................................................................Uagaucauugugaaaacuaauc...............   39    1   seq
.......................................................................gagaucauugugaaaacuaauc...............   286   0   seq
.......................................................................gagaucauugugaaaacCaauc...............   1     1   seq
.......................................................................gagaucauugugaaaacuaauA...............   2     1   seq
.......................................................................gagaucauuUugaaaacuaauc...............   1     1   seq
.......................................................................gagaucauugugUaaacuaauc...............   1     1   seq
.......................................................................gagaucauugugaaaacuaaucu..............   2     0   seq
........................................................................Ugaucauugugaaaacua..................   38    1   seq
........................................................................agaucauugugaaaacua..................   34    0   seq
........................................................................Ugaucauugugaaaacuaa.................   9     1   seq
........................................................................agaucauugugaaaacuaa.................   1     0   seq
........................................................................Ugaucauugugaaaacuaau................   54    1   seq
........................................................................Ggaucauugugaaaacuaau................   1     1   seq
........................................................................agaucauugugaaaacuaau................   11    0   seq
```

```
                                  Star                             Mature
ugcgaauguuucagagcaagacuugcgagacugguuuucucuuuggucuuccagauuuuuauuggaauucugagaucauugugaaaacuaaucucgcugacauuacgc
........................................................................agaucauugugaaaacuaauc...............   9    0    seq
........................................................................Ugaucauugugaaaacuaauc...............   59   1    seq
.........................................................................gaucauugugaaaacuaau................   5    0    seq
.........................................................................gaucauugugaaaacuaauc...............   5    0    seq
.........................................................................gauUauugugaaaacuaauc...............   1    1    seq
..........................................................................aucauugugaaaacuaauc...............   3    0    seq
```
